# Soil Microbial Functions Indicate Persistent Agricultural Legacies and Potential Alternative States Following Restoration Plantings

**DOI:** 10.1002/ece3.73172

**Published:** 2026-02-26

**Authors:** Shawn D. Peddle, Christian Cando‐Dumancela, Sofie Costin, Tarryn Davies, Michael P. Doane, Robert A. Edwards, Riley J. Hodgson, Siegfried L. Krauss, Craig Liddicoat, Martin F. Breed

**Affiliations:** ^1^ College of Science and Engineering Flinders University Bedford Park South Australia Australia; ^2^ The University of Western Australia Crawley Western Australia Australia; ^3^ Kings Park Science, Department of Biodiversity Conservation and Attractions Perth Western Australia Australia

**Keywords:** alternative stable states, functional capacity, land‐use legacy, metagenomics, microbiome, restoration, soil abiotic legacies, soil health

## Abstract

Soil microbiomes are fundamental ecosystem components that are increasingly used to monitor the efficacy of restoration efforts. However, given high levels of functional redundancy among soil microbial taxa and the subsequent lack of definitive taxa‐function links, taxonomic assessments (e.g., via metabarcoding) alone are limited for inferring ecological recovery. Here, we used shotgun metagenomics on soils from six post‐agricultural restoration sites in southwest Western Australia to test whether soil microbial functional potential recovers following restoration plantings. We compared taxonomic and functional gene diversity and composition across degraded, passively regenerated, revegetated, and remnant land conditions. Effective number of functions (alpha diversity) did not differ across land conditions. However, functional composition (beta diversity) differed between remnant and revegetated conditions and associated with altered soil abiotic properties, especially elevated phosphorus. Remnant soils supported a greater diversity of phosphorus metabolism functions despite lower available phosphorus, indicating a microbial adaptation to nutrient limitation in phosphorus deficient soils. Rather than indicating a lack of functional recovery, these results suggest a functional response to persistent agricultural legacies that may reflect a shift toward an alternative state. Restoration interventions that aim to target the soil microbiome (e.g., soil inoculations) or directly address abiotic legacies (e.g., phosphorus mining plants) may therefore be required to facilitate recovery of the soil microbial functions and the wider ecosystem.

## Graphical abstract

Soil microbial functions are key indicators of ecosystem recovery but can be obscured by taxonomic redundancy. Using shotgun metagenomics, we show that while overall functional diversity remains stable across land conditions, functional composition shifts persist in restored soils, particularly in relation to phosphorus metabolism. These findings suggest that agricultural legacies may maintain alternative states, and targeted interventions addressing soil microbiomes or abiotic legacies may be needed to fully restore ecosystem function.

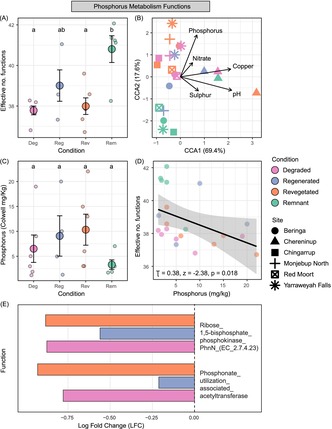

## 1 Introduction

The effective restoration of natural ecosystems on post‐agricultural land is important to address global biodiversity declines (Chazdon et al. 2020). However, post‐agricultural restoration often faces numerous challenges arising from altered soil abiotic properties including homogenised soil structure from tillage practices and soil nutrient legacies from decades of fertiliser application (Cramer et al. 2008; Brudvig et al. 2021). These post‐agricultural soil legacies can impede the recovery of both above‐ and below‐ground ecosystem components despite concerted restoration efforts (Suding et al. 2004; Turley et al. 2020). Assessing the recovery of soil microbial communities and their ecological functions following restoration provides an informative monitoring approach to assess the effectiveness of restoration interventions (Van Der Heyde et al. 2022; Robinson et al. 2023).

Soil microbial communities show mixed responses to restoration plantings. Some studies indicate a lack of recovery in microbial communities decades after restoration interventions (Turley et al. 2020; Peddle et al. 2024), while others more commonly indicate patterns toward recovery (Liddicoat et al. 2022; Barber et al. 2023), although recovery is often incomplete (Watson et al. 2022). While assessing the recovery of soil bacterial communities following post‐agricultural restoration sites in southwest Western Australia, Peddle et al. (2024) showed that agricultural land‐use legacies, particularly elevated levels of phosphorus, still persisted 17 years after the cessation of agricultural practices and revegetation efforts. These land‐use legacies also associated with a lack of recovery in soil bacterial community composition. While assessments of microbial communities can provide indications of ecological succession and recovery trajectories, a lack of recovery of community composition does not directly infer a lack of functional recovery due to high levels of functional redundancy (or functional similarity, see Eisenhauer et al. 2023) and horizontal gene transfer between taxa (Allison and Martiny 2008). These high levels of functional redundancy and the inability to directly link microbial function to taxonomy indicate that functional recovery could occur despite incomplete recovery of taxonomic composition.

Increasingly, studies are using single gene focused amplicon or eDNA metabarcoding approaches to assess the recovery of microbial communities following restoration (Mohr et al. 2022; Barber et al. 2023). However, because taxonomic identity alone cannot reliably predict microbial functional roles due to high functional redundancy and horizontal gene transfer (Allison and Martiny 2008; Nkongolo and Narendrula‐Kotha 2020), such approaches provide limited insight into the ecological processes underpinning recovery. Shotgun metagenomics overcomes these limitations by directly sequencing and quantifying microbial genomes, enabling more comprehensive assessments of soil functional capacity and microbially mediated processes such as nutrient cycling, organic matter decomposition, and primary productivity (Breed et al. 2019; Mason et al. 2023). Soil microbes drive these processes by converting organic matter, facilitating mineral weathering, and mobilising key nutrients like nitrogen and phosphorus required by plants and other organisms (Cavicchioli et al. 2019; Pang et al. 2024). In nutrient‐poor systems such as those of southwest Western Australia, microbial functional diversity is especially critical, supporting plant–microbe interactions and enabling adaptation to phosphorus limitation (Yao et al. 2018; Oliverio et al. 2020). Metagenomic approaches therefore provide powerful tools to evaluate ecosystem recovery and resilience by revealing how microbial functional potential responds to restoration interventions and persistent soil legacies (Robinson et al. 2023; Peddle et al. 2025).

Here, we used shotgun metagenomics to characterise the diversity and composition of soil microbial communities and their functions across four land conditions (degraded, passive regenerated, active revegetated, remnant) in six post‐agricultural restoration sites in southwest Western Australia. We specifically tested the following hypotheses: (i) Microbial taxonomic diversity and composition differ among land conditions, reflecting persistent effects of agricultural legacies and partial recovery following restoration; (ii) The composition and diversity of microbial functional genes differ among land conditions, reflecting functional responses to persistent soil legacies following agricultural land use; (iii) Functions associated with phosphorus cycling show distinct patterns of recovery across land conditions, reflecting both nutrient legacies and microbially mediated responses to altered phosphorus availability. Although previous amplicon‐based assessments of the recovery of bacterial communities at these sites indicated a lack of recovery of community composition (Peddle et al. 2024), assessing the recovery of microbial functions—such as phosphorus metabolism—will be valuable for evaluating recovery trajectories, efficacy of restoration interventions, and any corresponding shifts in functional resilience (Mason et al. 2023).

## 2 Materials and Methods

### 2.1 Site Description and Soil Sampling

**FIGURE 1:**
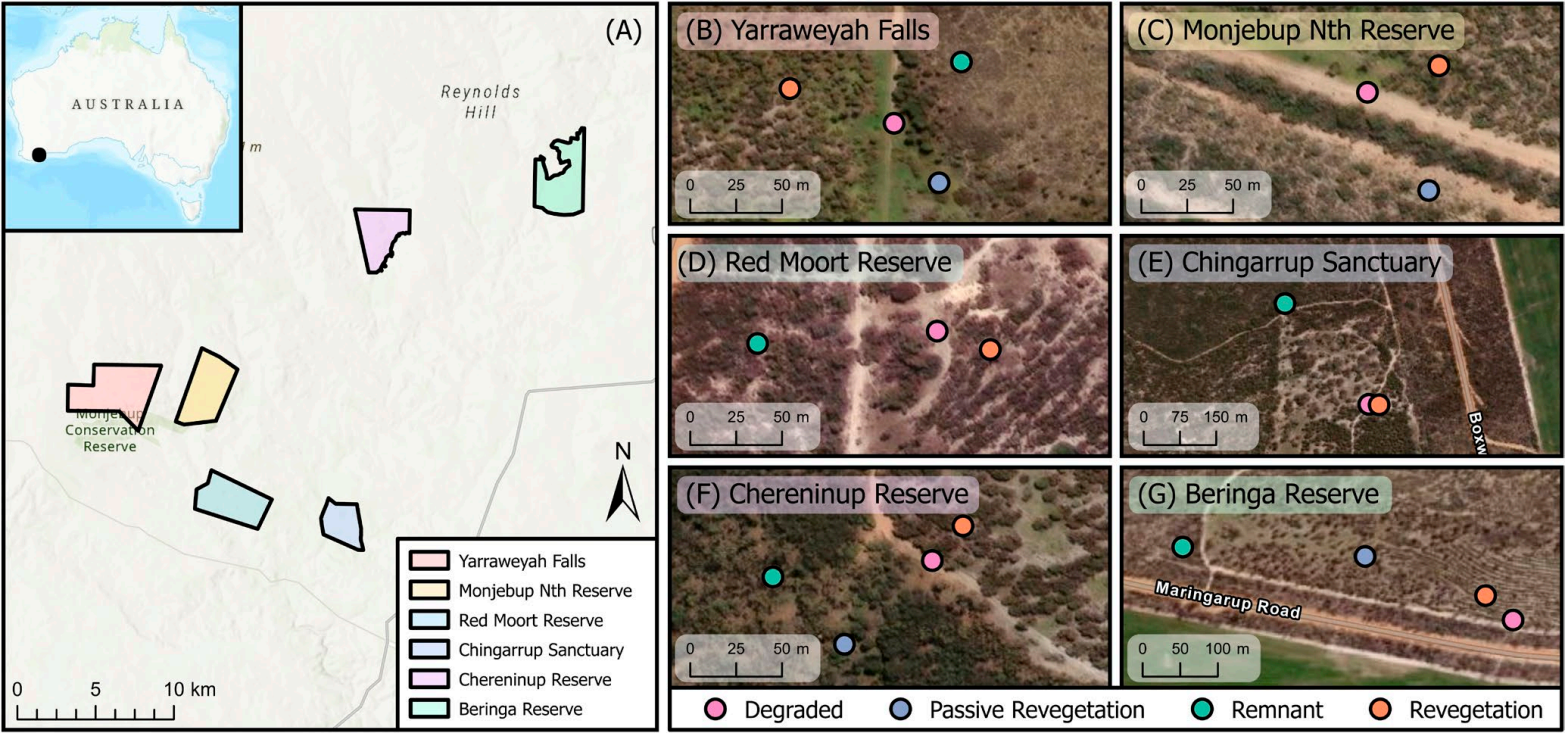
Maps indicating (A) location of the six study sites used for soil sampling in southwest Western Australia; and sampling design and layout of degraded, passive restoration, revegetation, and remnant land conditions at each sampled site (B) Yarraweyah Falls, (C) Monjebup North Reserve, (D) Red Moort Reserve, (E) Chingarrup Sanctuary, (F) Chereninup Reserve, (G) Beringa Reserve.

This study was conducted at six sites in southwest Western Australia (Yarraweyah Falls, Monjebup Reserve, Red Moort Reserve, Chingarrup Sanctuary, Chereninup Reserve, and Beringa Reserve), situated between the Stirling Range National Park and Fitzgerald River National Park (Figure 1). While all sites are currently managed for conservation, they were each previously used for agricultural cropping and experienced substantial clearing of native vegetation. The region falls within the southwest Australian floristic zone, recognised as a global biodiversity hotspot due to its extraordinary plant species richness, high levels of endemism, and extensive habitat fragmentation resulting from land clearing (Myers et al. 2000; Gioia and Hopper 2017). To counteract this, various conservation groups are working to restore ecological connectivity by re‐establishing a diverse mallee heath vegetation community as part of the Gondwana Link project (Bradby et al. 2016). The region experiences a Mediterranean climate, with hot, dry summers and cool, wet winters, and receives an average annual rainfall of approximately 455 mm (Australian Bureau of Meteorology 2025). The soils are typically nutrient‐poor and range from shallow to deep sandy duplexes, characterised by sandy or sandy‐loam topsoils overlaying clay or heavier‐textured subsoils.

Each of the six sites was largely cleared for agricultural cropping and grazing in the mid‐20th century and has since undergone extensive revegetation between 2002 and 2017 using species‐rich seed mixes sourced from local provenances. In this study, four distinct land conditions were identified within each site (hereafter referred to as *land condition*, denoting site status along a degradation–restoration gradient rather than an experimental treatment): (i) remnant natural vegetation that was never cleared or used for cultivation (hereafter, remnant), (ii) historically cleared areas with minimal to no agricultural use that passively regenerated without direct seeding or planting (hereafter, regenerated), (iii) formerly cleared agricultural land that has been actively revegetated via direct seeding (hereafter, revegetated), and (iv) degraded areas that were historically cleared for agriculture and are now actively managed to suppress vegetation for firebreaks or access tracks (hereafter, degraded). A sampling design was implemented to ensure each site contained all four land conditions in close proximity (Figure 1). At each site, 25 × 25 m quadrats were established within each of the four land conditions in September 2020. Within each of these quadrats, nine soil subsamples were systematically collected from the top 10 cm of topsoil to account for plot‐scale spatial heterogeneity. These subsamples were pooled and homogenised, with a 50 mL sample frozen on‐site for subsequent DNA extraction and 500 g of soil retained for physicochemical analysis at CSBP Laboratories (Perth, Western Australia) quantifying texture, pH, conductivity, organic carbon, available phosphorus (Colwell), ammonium nitrogen, nitrate nitrogen, potassium (Colwell), sulphur, and total copper, iron, manganese, zinc, aluminium, calcium, sodium, and boron.

### 2.2 Shotgun Metagenomic Sequencing and Bioinformatics

DNA was extracted from each soil sample using the Qiagen DNeasy Powerlyzer Powersoil Kit (Qiagen, Hilden, Germany) following the manufacturer's instructions and quantified fluorometrically. Libraries were prepared using Nextera library prep kits (Illumina, San Diego, CA); then linear DNA libraries were converted into DNA nanoball (DNB) structures using the MGIEasy Universal Library Conversion Kit (MGI, China). Three of our samples failed library prep, and the remaining 21 libraries were sequenced at the South Australian Genomics Centre (SAGC) with the MGI DNBSEQ G400 (MGI, China) producing 2 × 150 bp paired‐end sequences. Bioinformatic processing of the metagenomics data was performed using DeepThought high performance computing (Flinders University 2021). Data cleaning was conducted using fastp v0.23.2 (Chen et al. 2018), which included trimming adapters from DNB sequences. Taxonomic IDs were assigned using Kraken2 v2.0.7 (Wood et al. 2019) using the PlusPFP database (k2_pluspfp_20231009, downloaded 9 October 2023), followed by Bracken v2.9 (Lu et al. 2017) to estimate taxonomic abundances at the species level using a read length of 100 bp and the same database. Bracken reports were converted to MeataPhlAn‐style profiles using KrakenTools (Lu et al. 2022) and combined into a single taxonomic abundance table. We assigned prokaryotic gene functions directly to quality‐filtered reads using SUPER‐FOCUS v1.6 (Silva et al. 2015) with the Diamond v0.9.14 aligner (Buchfink et al. 2021). We used a read‐based approach because assembly in highly diverse soil metagenomes can bias against low‐abundance genes and obscure community‐wide functional capacity, whereas direct read annotation preserves functional diversity and is widely applied in soil ecosystem studies (Sun and Badgley 2019; Mason et al. 2023). Functional assignments were subsequently grouped into unique functional processes and three thematic functional subsystems from the DB_100 SEED database (Overbeek et al. 2004).

### 2.3 Statistical Analysis

All statistical analyses were conducted in R version 4.4.0 (R Core Team 2024). Analyses were designed to assess differences in microbial taxonomic and functional diversity and composition among land conditions and sites, and to evaluate associations between compositions and soil physicochemical properties. Unless stated otherwise, identical analytical frameworks were applied to taxonomic data, the full functional gene dataset, and targeted functional subsystems. Relative abundance normalisation was applied to both taxonomic and functional datasets to account for differences in sequencing depth among samples. Because SUPER‐FOCUS assigns functions only to prokaryotic reads, taxa classified as Eukaryote at the kingdom level were removed from the taxonomic dataset to ensure comparability between taxonomic and functional analyses. Soil physicochemical variables were mean‐centred and standardised prior to multivariate analyses.

#### 2.3.1 Alpha Diversity

To test hypotheses H1 and H2, we quantified and compared taxonomic and functional alpha diversity among land conditions. Alpha diversity was quantified as the effective number of species or functions, calculated as the exponent of Shannon's diversity index (Jost 2006). Differences among land conditions were tested using analysis of variance (ANOVA) with Tukey post hoc comparisons. Assumptions of all ANOVA models were checked on residuals using Shapiro–Wilk tests for normality, Q–Q plots, and Bartlett tests for homogeneity of variances. This approach was applied to taxonomic diversity, overall functional diversity, and selected functional subsets.

#### 2.3.2 Community and Functional Composition

To test hypotheses H1 and H2, we examined differences in microbial taxonomic and functional composition among land conditions and sites. Differences in microbial taxonomic and functional composition were visualised using non‐metric multidimensional scaling (NMDS) ordinations based on Bray–Curtis dissimilarities. Statistical differences in composition among land conditions and sites were tested using permutational multivariate analysis of variance (PERMANOVA) with the adonis2 function in the vegan package (Oksanen et al. 2013), using 999 permutations. These analyses were applied consistently to taxonomic composition, overall functional composition, and selected functional subsets.

#### 2.3.3 Associations With Soil Physicochemical Properties

To contextualise hypotheses H1 and H2, we assessed associations between community and functional composition and soil physicochemical properties. Associations between microbial community or functional composition and soil physicochemical properties were assessed using constrained correspondence analysis (CCA). Prior to analysis, highly collinear soil variables (*r* > 0.75) were removed using the *findCorrelation* function in the *caret* package (Kuhn 2015). Final models were selected using forward stepwise selection (*ordistep* in *vegan*), and the significance of constrained models and individual axes was assessed using permuted ANOVA with 999 permutations. This analytical framework was applied to taxonomic composition, overall functional composition, and selected functional subsets.

#### 2.3.4 Functional Group Analyses

To directly test hypothesis H3, we examined patterns in functional groups associated with phosphorus cycling and other nutrient‐related processes across land conditions. To characterise patterns across the functional dataset, the relative abundance of the 20 most abundant subsystem 1 functional categories were visualised using stacked bar plots. Differences in relative abundance among land conditions were tested using permuted ANOVAs. Functional groups showing significant differences among land conditions, as well as those linked to previously identified abiotic legacies (i.e., phosphorus metabolism, iron acquisition and metabolism, sulphur acquisition and metabolism, and nitrogen metabolism), were selected for further analysis.

For these selected functional groups, alpha diversity, compositional differences, and associations with soil physicochemical properties were assessed using the same analytical frameworks described above. Relative abundances of finer‐scale functional categories (subsystem 2 or subsystem 3, where applicable) were visualised and tested individually for differences among land conditions using permuted ANOVAs.

#### 2.3.5 Differential Abundance and Correlation Analyses

Differential abundance and correlation analyses were used to further resolve functional differences related to phosphorus cycling (H3). To identify specific phosphorus metabolism functions contributing to compositional differences among land conditions, differential abundance analyses were conducted using ANCOMBC2 on raw count data (Lin and Peddada 2024). Pairwise contrasts were performed comparing degraded, regenerated, and revegetated soils against remnant soils.

Relationships between phosphorus metabolism functional diversity and available soil phosphorus were assessed using Kendall's rank correlation. Differences in available phosphorus among land conditions were tested using ANOVA and visualised using scatterplots.

## 3 Results

Our analysis generated taxonomic libraries with a total of 64,345,215 reads (mean 3,064,058 reads per sample) containing 13,940 unique microbial species (mean 10,344 per sample) (Table S1). Our functional libraries consisted of 72,657,207 reads (mean 3,459,867 per sample) containing 35,276 unique functions (mean 24,021 per sample) (Table S1).

### 3.1 Alpha Diversity

**FIGURE 2:**
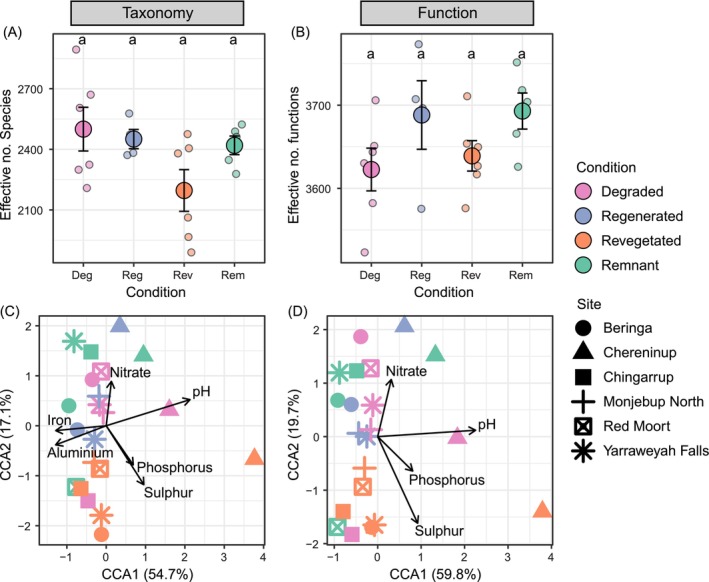
Taxonomic (A, C) and Functional (B, D) diversities, compositions and associations with soil abiotic properties. (A) Effective number of species (mean ± SE) in soil samples collected across four land conditions. Groups sharing the same letter do not differ (*p* > 0.05). (B) Effective number of functions (mean ± SE) in soil samples collected across four land conditions. Groups sharing the same letter do not statistically differ (*p* > 0.05). (C) Constrained correspondence analysis (CCA) indicating microbial taxonomic compositions and the associated model‐selected soil abiotic properties. (D) Constrained correspondence analysis (CCA) indicating the compositions of microbial functions and the associated model‐selected soil abiotic properties.

The effective number of species did not differ among our four land conditions (Figure 2A, Table S2; ANOVA, df = 3, *F* = 2.42, *p* = 0.102), although revegetated soil exhibited the lowest effective number of species (mean effective no. species ± SD: revegetated 2196.32 ± 252.89, remnant 2419.69 ± 102.89, regenerated 2450.89 ± 95.28, degraded 2500.00 ± 265.11). Across the full functional dataset, effective number of functions also did not differ among land conditions (Figure 2B; ANOVA, df = 3, *F* = 1.841, *p* = 0.178).

### 3.2 Community and Functional Composition

**FIGURE 3:**
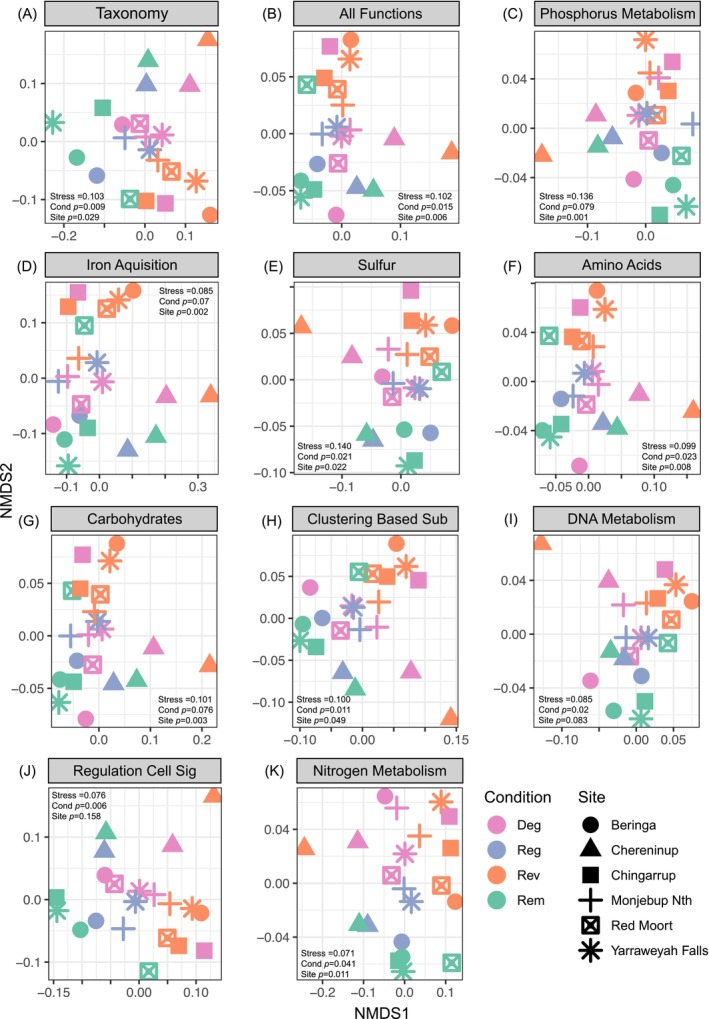
Non‐metric multidimensional scaling ordinations (NMDS) indicating compositions of microbial (A) Taxonomy, (B) All functions, (C) Phosphorus metabolism, (D) Iron acquisition and metabolism, (E) Sulphur acquisition and metabolism, (F) Amino Acids and Derivates, (G) Carbohydrates, (H) Clustering‐based subsystems, (I) DNA metabolism, (J) Regulation and cell signalling, (K) Nitrogen metabolism functions across the four land conditions and six sites. Differences (*p* < 0.05) across land condition and site were tested with PERMANOVA.

Microbial taxonomic composition varied across both land condition and site (Table S3, Figure 3A; PERMANOVA, condition, *F*_3,17_ = 2.242, *R* ^2^ = 0.283, *p* = 0.009; site, *F*_5,15_ = 1.703, *R* ^2^ = 0.362, *p* = 0.029). We also found compositional differences when comparing all functions represented in samples across land condition and site (Table S3, Figure 3B; PERMANOVA, condition, *F*_3,17_ = 2.015, *R* ^2^ = 0.262, *p* = 0.015; site, *F*_5,15_ = 1.977, *R* ^2^ = 0.397, *p* = 0.006), with remnant and revegetated conditions displaying clear differences in functional compositions.

### 3.3 Associations With Soil Physicochemical Properties

After removing highly correlated variables, we found associations between microbial community composition and nitrate, pH, phosphorus, sulphur, aluminium, and iron (Figure 2C). CCA also showed associations between functional compositions across land conditions with nitrate, pH, phosphorus and sulphur (Figure 2D).

#### 3.3.1 Functional Group Analyses

We identified 35 functional groups (or themes) at the subsystem 1 level, the 20 most abundant of which represented 99.00% of the sum total functional capacity attributed across all samples (Figure S1). The relative abundance of functions related to amino acids and derivatives (permuted ANOVA, *t* = 9.588, BH adj *p* = 0.034), carbohydrates (permuted ANOVA, *t* = 9.905, BH adj *p* = 0.034), clustering‐based subsystems (permuted ANOVA, *t* = 11.230, BH adj *p* = 0.020), DNA metabolism (permuted ANOVA, *t* = 13.217, BH adj *p* = 0.002), and regulation and cell signalling (permuted ANOVA, *t* = 11.688, BH adj *p* = 0.019) differed across land conditions (Figure S1) and therefore were examined in more detail (see below), along with phosphorus metabolism, iron acquisition and metabolism, sulphur acquisition and metabolism, and nitrogen metabolism functions (as previously noted).

**FIGURE 4:**
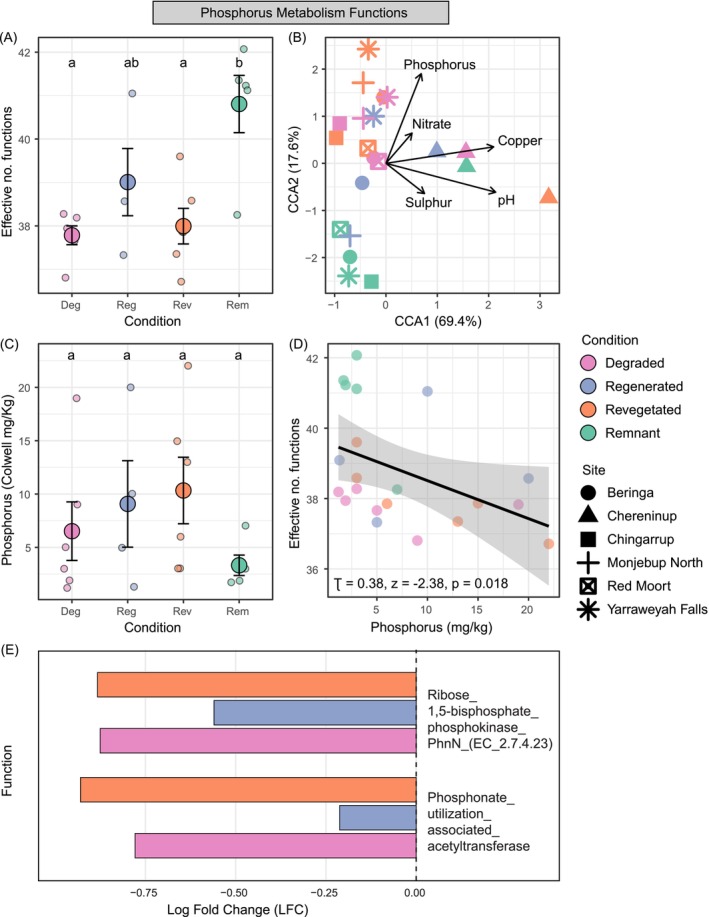
Functional diversity, composition and associations with soil abiotic properties for phosphorus metabolism functions at the functional process level. (A) Effective number of phosphorus metabolism functions (mean ± SE) in soil samples collected across four land conditions. Groups sharing the same letter do not differ (*p* > 0.05, Kruskal‐Wallis, Dunn test). (B) Constrained correspondence analysis (CCA) indicating the composition of phosphorus metabolism functions and the associated model‐selected soil abiotic properties. (C) Phosphorus (Colwell) levels (mean ± SE) in soil samples collected across the four land conditions. Groups sharing a letter do not differ (*p* > 0.05, Kruskal‐Wallis, Dunn test). (D) Correlation (Kendall's Tau) of effective number of phosphorus metabolism functions and soil phosphorus levels. (E) Bar plot of significant differential abundances (log fold change *p* < 0.05) in phosphorus metabolism functions with pairwise comparisons of degraded (Deg), regenerated (Reg) and revegetated (Rev) land conditions each to the remnant condition.

For phosphorus metabolism, remnant samples had the highest effective number of phosphorus metabolism functions and were significantly greater than the effective number of phosphorus metabolism functions from degraded and revegetated land conditions (Table S2, Figure 4A; ANOVA, df = 3, *F* = 7.722, *p* = 0.001). While we only found weak evidence that the composition of phosphorus metabolism functions differed across land condition, they differed across site (Table S3, Figure 3C; PERMANOVA, condition, *F*_3,17_ = 1.683, *R* ^2^ = 0.229, *p* = 0.079; site, *F*_5,15_ = 2.917, *R* ^2^ = 0.493, *p* = 0.001). CCA indicated phosphorus functional compositions across land condition associated with phosphorus, nitrate, copper, pH, and sulphur (Figure 4B).

**FIGURE 5:**
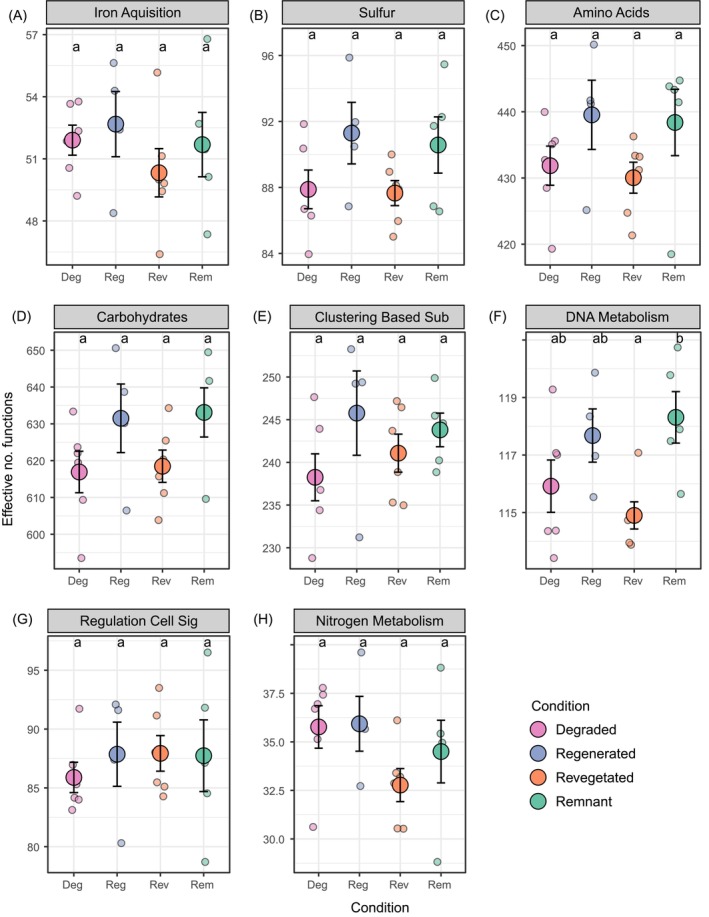
Effective number of functions (mean ± SE) for all remaining subsystem 1 level functions (A) Iron, (B) Sulphur acquisition and metabolism, (C) Amino Acids and Derivates, (D) Carbohydrates, (E) Clustering‐based subsystems, (F) DNA metabolism, (G) Regulation and cell signalling, (H) Nitrogen metabolism. Different letters across Land condition indicate significant (*p* < 0.05) differences.

**FIGURE 6:**
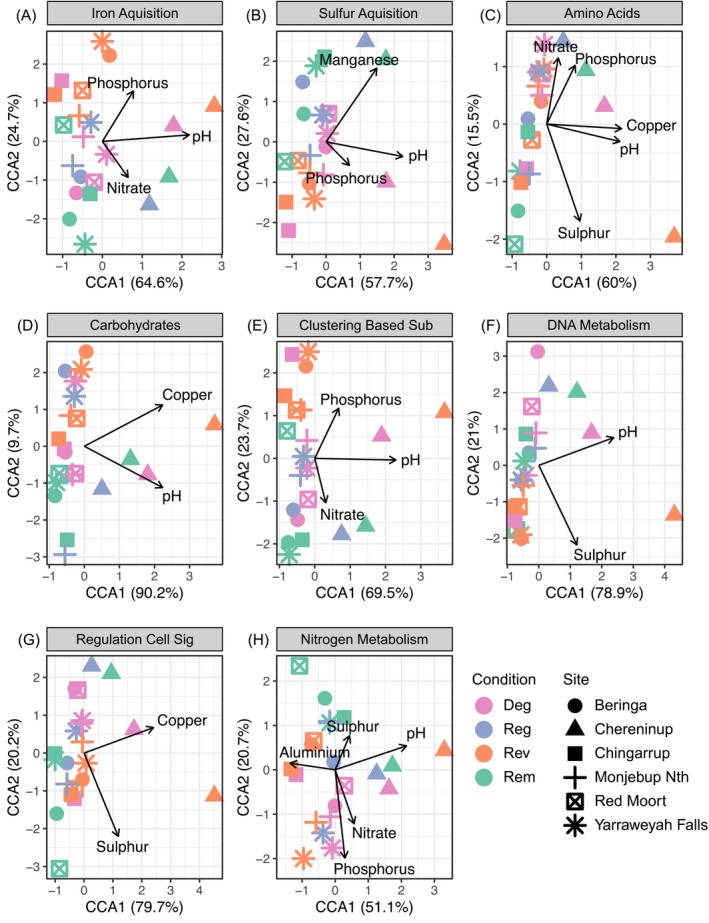
Constrained correspondence analyses of functional compositions for all remaining assessed subsystem 2 level functions (A) Iron acquisition and metabolism, (B) Sulphur acquisition and metabolism, (C) Amino Acids and Derivates, (D) Carbohydrates, (E) Clustering‐based subsystems, (F) DNA metabolism, (G) Regulation and cell signalling, (H) Nitrogen metabolism and their significant associations with measured physicochemical properties.

For all the other subsystem 1 functional groups we assessed (i.e., amino acids and derivatives, carbohydrates, clustering‐based subsystems, DNA metabolism, regulation and cell signalling, iron acquisition and metabolism, and sulphur acquisition and metabolism), only the DNA metabolism functional group differed in effective number of functions with the remnant land condition differing to revegetation (Figure 5; Tukey, diff = 3.41, 95% CI [0.23, 6.59] adj *p* = 0.03, remnant 118.31 ± 2.00SD, regenerated 117.68 ± 1.85, degraded 115.92 ± 2.23, revegetated 114.90 ± 1.16). The compositions of functions for most subsystem groups differed across land conditions and sites (Table S3, Figure 3). Although functional compositions in all other subsystem groups associated with various soil abiotic properties, specific trends of associations across land conditions were less clear than the associations with compositions at one site in particular (i.e., Chereninup; see Figure 6). For the relative abundance of subsystem 2 or 3 groups within each of the assessed subsystem 1 groups, few groups differed across land condition (Figures S3–S10).

### 3.4 Differential Abundance and Correlation Analyses

We found no difference in the relative abundance of any specific phosphorus metabolism subsystem 3 functional group across land conditions (Figure S2; permuted ANOVA, *p* > 0.05 for all). Two phosphorus metabolism functions—Phosphonate utilisation associated acetyltransferase and Ribose 1,5 bisphosphate phosphokinase (PhnN; EC 2.7.4.23)—showed condition‐specific reduced abundances relative to the remnant condition (Figure 4E, Table S4).

While available phosphorus (Colwell) levels did not differ statistically (likely due to low sample sizes) across our land conditions (Figure 4C; ANOVA, df = 3, *F* = 1.164, *p* = 0.352), phosphorus levels were generally lower in remnant soils and 2–3 times higher in revegetated soils (remnant 3.32 ± 2.14, degraded 6.52 ± 6.73, regenerated 9.07 ± 8.11, revegetated 10.3 ± 7.63). Kendall's rank correlation test showed a moderate negative correlation between phosphorus levels and phosphorus metabolism functional diversity (Figure 4D; *τ* = −0.38, *z* = −2.38, *p* = 0.018).

## 4 Discussion

Restoring soil microbial function is a major challenge in post‐agricultural landscapes. Using metagenomics on soils from southwest Western Australia, we found that functional alpha diversity was consistent across degraded, regenerated, revegetated, and remnant land conditions, but functional composition differed, particularly between revegetated and remnant soils. These compositional differences were strongly linked to soil abiotic legacies, especially phosphorus availability. Remnant soils supported the greatest diversity of phosphorus metabolism functions despite the lowest levels of available phosphorus, reflecting microbial adaptation to phosphorus cycling under nutrient limitation. Taxonomic differences observed across land conditions were expressed primarily as shifts in overall community composition rather than consistent changes in the relative abundance of specific higher‐level taxa. Together, these findings suggest that persistent agricultural legacies—particularly altered phosphorus levels from fertiliser application—shape microbial functional trajectories and may promote the emergence or persistence of an alternative state. Targeted interventions, such as soil inoculations to initiate microbial recovery or phosphorus reduction through the introduction of phosphorus mining plants, may therefore be required to overcome these legacies and achieve effective restoration outcomes (Peddle et al. 2025).

The greater diversity of phosphorus metabolism functions in remnant soils with low available phosphorus suggests long‐term selection for microbial communities with a broader functional potential for phosphorus acquisition under nutrient limitation. This pattern is supported by the negative correlation between phosphorus availability and phosphorus functional diversity. In remnant soils, community‐level enrichment of diverse phosphorus cycling genes likely reflects the persistence of multiple phosphorus acquisition strategies among co‐occurring taxa rather than increased rates of functional activity (Yao et al. 2018; Oliverio et al. 2020). In contrast, revegetated and degraded soils with elevated phosphorus showed reduced diversity in these pathways, consistent with preferential use of inorganic phosphate over phosphonates, which requires less energy investment (Condron et al. 2005). Although compositional differences in phosphorus functions were modest, they were strongest between remnant and revegetated soils and aligned with abiotic differences, particularly lower phosphorus in remnants. Moreover, the repeated associations of soil phosphorus levels with the compositions (beta diversity) of multiple other functional subsystems, as well as to taxonomic beta diversity, provides further evidence for the strong role of post‐agricultural phosphorus legacies impacting the recovery of microbial communities and their functions (Peddle et al. 2024; Wang et al. 2024). Our findings align with previous research indicating that phosphorus availability is a key soil property that shapes microbial and functional compositions, particularly in dry and phosphorus‐limited soil environments (Liu et al. 2012; Delgado‐Baquerizo et al. 2017; Oliverio et al. 2020).

Although we did not identify any differences in the relative abundances of subsystem 3 phosphorus metabolism levels (e.g., Alkylphosphonate utilisation, P uptake cyanobacteria, high affinity phosphate transporter, control of PHO regulon etc.), two specific phosphorus metabolism genes at the functional process level (Phosphonate utilisation associated acetyltransferase and Ribose 1,5 bisphosphate phosphokinase PhnN EC) involved with microbial degradation of phosphonates were less abundant in revegetated, degraded, and, to a lesser extent, regenerated soils compared to remnant soils. Agricultural soils in Western Australia have a history of extensive application of superphosphate fertiliser, which can lead to a legacy of elevated phosphorus even decades after revegetation and restoration (Lambers et al. 2011; Daws et al. 2019; Parkhurst, Standish, and Prober 2022). The decreased abundance of these two genes in revegetated post‐agricultural soils likely reflects a shift away from low phosphorus microbial acquisition strategies due to higher residual phosphorus. Together, these findings indicate that while overall phosphorus functional diversity appears highest in remnant soils, the specific metabolic pathways utilised by microbial communities differ in post‐agricultural land conditions, potentially influencing phosphorus cycling dynamics and long‐term ecosystem recovery.

We show that several abiotic soil properties strongly associate with microbial community and functional compositions, with nitrate, pH, phosphorus, sulphur, aluminium, and iron each associating with either microbial community or functional compositions. Some of these multivariate associations were particularly pronounced at the Chereninup site, likely reflecting site‐specific soil heterogeneity, but the direction and nature of these relationships were consistent with the broader patterns observed across land conditions. These findings reinforce the importance of soil abiotic properties in shaping restoration outcomes (Robinson, Liddicoat, et al. 2024) highlighting that outcomes are not solely dictated by vegetation establishment but are also potentially constrained by soil legacies from past land use (Toledo et al. 2018; Parkhurst, Prober, et al. 2022; Peddle et al. 2024). While overall functional diversity did not differ across our land conditions, subtle compositional shifts indicate that functions in revegetated soils remain distinct from those in remnant ecosystems. These subtle differences in functions do not necessarily reflect a ‘lack of recovery’ or dysfunction per se but may instead reflect altered functional dynamics consistent with the emergence of an alternative state in restoration sites with unaddressed land‐use legacies (Suding et al. 2004). We acknowledge that our single time‐point sampling design cannot directly demonstrate temporal stability, a key criterion of alternative stable states. However, the persistent compositional and functional differences aligned with altered soil phosphorus conditions are consistent with the mechanisms proposed to underlie alternative states in terrestrial ecosystems.

If full functional recovery to match remnant conditions is the goal of a restoration project, addressing these constraints through targeted soil translocations/inoculations and/or biostimulation strategies (e.g., ecological phage therapy, Davies et al. 2024; sonic restoration, Robinson, Annells, et al. 2024) may be beneficial to accelerate microbial functional recovery in soil ecosystems. Moreover, further research of functional recovery using RNA‐based metatranscriptomic sequencing is needed to confirm if these functions are being actively expressed in the environment (Breed et al. 2019). The metagenomic sequencing methods we use here are limited in that they can only be used infer functional potential from the presence of genes, not their active expression (Cordier et al. 2019). Although, we would expect the functional capacities of microbiota to be shaped by available resources and environmental conditions over extended periods of time (Zhu et al. 2023). Furthermore, while our analyses focused on prokaryotic communities, fungi and other soil eukaryotes are known to play critical roles in soil structure, nutrient cycling, and restoration trajectories (Aslani et al. 2025), highlighting an important avenue for future work integrating multi‐kingdom approaches.

The strong association between phosphorus metabolism diversity and available phosphorus levels indicates that post‐agricultural abiotic legacies continue to shape microbial functions, constraining long‐term nutrient cycling and plant‐microbe interactions. Accordingly, restoration efforts should not only focus on vegetation recovery but also on strategies to promote microbial functional recovery that, for example, supports ecosystem processes such as nutrient cycling. Interventions such as soil inoculations with microbial communities sourced from remnant ecosystems, or phosphorus mining from soil via phosphorus accumulating plants (Ryan et al. 2009), may help restore pre‐disturbance soil conditions and functional compositions. However, the effectiveness of such approaches will depend on microbial establishment and persistence despite altered soil conditions and the development of practical techniques to reduce available phosphorus in soil. These uncertainties highlight the need for further research on how to overcome land‐use legacies and to identify the ecological and abiotic constraints on microbial functional recovery in post‐agricultural landscapes, consistent with findings across a range of terrestrial landscapes (Peddle et al. 2024; Gomes et al. 2025).

## Author Contributions

**Shawn D. Peddle:** conceptualization (lead), data curation (lead), formal analysis (lead), investigation (lead), writing – original draft (lead), writing – review and editing (lead). **Christian Cando‐Dumancela:** data curation (supporting), writing – review and editing (supporting). **Sofie Costin:** resources (supporting), writing – review and editing (supporting). **Tarryn Davies:** resources (supporting), writing – review and editing (supporting). **Michael P. Doane:** resources (supporting), writing – review and editing (supporting). **Robert A. Edwards:** resources (supporting), supervision (supporting), writing – review and editing (supporting). **Riley J. Hodgson:** formal analysis (supporting), writing – review and editing (supporting). **Siegfried L. Krauss:** conceptualization (supporting), resources (equal), writing – review and editing (supporting). **Craig Liddicoat:** formal analysis (supporting), supervision (equal), writing – review and editing (supporting). **Martin F. Breed:** conceptualization (equal), funding acquisition (lead), resources (equal), supervision (lead), writing – review and editing (equal).

## Funding

This work was supported by the Australian Research Council (LP190100051 and LP240100073), the New Zealand Ministry of Business Innovation and Employment (UOWX2101) and the Holsworth Wildlife Research Endowment.

## Conflicts of Interest

The authors declare no conflicts of interest.

## Supporting information


**Data S1:** ece373172‐sup‐0001‐Supinfo.docx.

## Data Availability

The raw metagenomic data generated for this project are available under BioProject PRJNA788638, accession numbers SRR17318367 through SRR17318387. All R code used for analysis is available on figshare, https://doi.org/10.6084/m9.figshare.30091063.

## References

[ece373172-bib-0001] Allison, S. D. , and J. B. H. Martiny . 2008. “Resistance, Resilience, and Redundancy in Microbial Communities.” Proceedings of the National Academy of Sciences of the United States of America 105: 11512–11519. 18695234 10.1073/pnas.0801925105 PMC2556421

[ece373172-bib-0002] Aslani, F. , L. Tedersoo , S. I. F. Gomes , S. Põlme , S. Anslan , and T. M. Bezemer . 2025. “Shifting Community Assembly Mechanisms in Soil Eukaryotes Over Successional Time and Their Implications for Restoration.” Scientific Reports 15: 25804. 40670560 10.1038/s41598-025-11507-8 PMC12267841

[ece373172-bib-0003] Australian Bureau of Meteorology . 2025. “Climate Statistics for Australian Locations.” http://www.bom.gov.au/jsp/ncc/cdio/weatherData/av?p_nccObsCode=139&p_display_type=dataFile&p_stn_num=010897.

[ece373172-bib-0004] Barber, N. A. , D. M. Klimek , J. K. Bell , and W. D. Swingley . 2023. “Restoration Age and Reintroduced Bison May Shape Soil Bacterial Communities in Restored Tallgrass Prairies.” FEMS Microbiology Ecology 99: fiad007. 36669763 10.1093/femsec/fiad007

[ece373172-bib-0005] Bradby, K. , A. Keesing , and G. Wardell‐Johnson . 2016. “Gondwana Link: Connecting People, Landscapes, and Livelihoods Across Southwestern Australia.” Restoration Ecology 24: 827–835.

[ece373172-bib-0006] Breed, M. F. , P. A. Harrison , C. Blyth , et al. 2019. “The Potential of Genomics for Restoring Ecosystems and Biodiversity.” Nature Reviews Genetics 20: 615–628. 10.1038/s41576-019-0152-0 31300751

[ece373172-bib-0007] Brudvig, L. A. , N. E. Turley , S. L. Bartel , et al. 2021. “Large Ecosystem‐Scale Effects of Restoration Fail to Mitigate Impacts of Land‐Use Legacies in Longleaf Pine Savannas.” Proceedings of the National Academy of Sciences of the United States of America 118: e2020935118. 33875596 10.1073/pnas.2020935118 PMC8092381

[ece373172-bib-0008] Buchfink, B. , K. Reuter , and H.‐G. Drost . 2021. “Sensitive Protein Alignments at Tree‐Of‐Life Scale Using DIAMOND.” Nature Methods 18: 366–368. 33828273 10.1038/s41592-021-01101-x PMC8026399

[ece373172-bib-0009] Cavicchioli, R. , W. J. Ripple , K. N. Timmis , et al. 2019. “Scientists' Warning to Humanity: Microorganisms and Climate Change.” Nature Reviews Microbiology 17: 569–586. 31213707 10.1038/s41579-019-0222-5 PMC7136171

[ece373172-bib-0010] Chazdon, R. L. , D. Lindenmayer , M. R. Guariguata , R. Crouzeilles , J. M. R. Benayas , and E. L. Chavero . 2020. “Fostering Natural Forest Regeneration on Former Agricultural Land Through Economic and Policy Interventions.” Environmental Research Letters 15: 043002.

[ece373172-bib-0011] Chen, S. , Y. Zhou , Y. Chen , and J. Gu . 2018. “Fastp: An Ultra‐Fast All‐In‐One FASTQ Preprocessor.” Bioinformatics 34: i884–i890. 30423086 10.1093/bioinformatics/bty560 PMC6129281

[ece373172-bib-0012] Condron, L. M. , B. L. Turner , and B. J. Cade‐Menun . 2005. “Chemistry and Dynamics of Soil Organic Phosphorus.” In Phosphorus: Agriculture and the Environment, 87–121. American Society of Agronomy.

[ece373172-bib-0013] Cordier, T. , A. Lanzén , L. Apothéloz‐Perret‐Gentil , T. Stoeck , and J. Pawlowski . 2019. “Embracing Environmental Genomics and Machine Learning for Routine Biomonitoring.” Trends in Microbiology 27: 387–397. 30554770 10.1016/j.tim.2018.10.012

[ece373172-bib-0014] Cramer, V. A. , R. J. Hobbs , and R. J. Standish . 2008. “What's New About Old Fields? Land Abandonment and Ecosystem Assembly.” Trends in Ecology & Evolution 23: 104–112. 18191278 10.1016/j.tree.2007.10.005

[ece373172-bib-0015] Davies, T. , C. Cando‐Dumancela , C. Liddicoat , et al. 2024. “Ecological Phage Therapy: Can Bacteriophages Help Rapidly Restore the Soil Microbiome?” Ecology and Evolution 14: e70185. 39145040 10.1002/ece3.70185 PMC11322231

[ece373172-bib-0016] Daws, M. I. , A. H. Grigg , M. Tibbett , and R. J. Standish . 2019. “Enduring Effects of Large Legumes and Phosphorus Fertiliser on Jarrah Forest Restoration 15 Years After Bauxite Mining.” Forest Ecology and Management 438: 204–214.

[ece373172-bib-0017] Delgado‐Baquerizo, M. , P. B. Reich , A. N. Khachane , et al. 2017. “It Is Elemental: Soil Nutrient Stoichiometry Drives Bacterial Diversity.” Environmental Microbiology 19: 1176–1188. 27943556 10.1111/1462-2920.13642

[ece373172-bib-0018] Eisenhauer, N. , J. Hines , F. T. Maestre , and M. C. Rillig . 2023. “Reconsidering Functional Redundancy in Biodiversity Research.” npj Biodiversity 2: 9. 39242717 10.1038/s44185-023-00015-5 PMC11332098

[ece373172-bib-0019] Flinders University . 2021. “Deep Thought (HPC).” 10.25957/FLINDERS.HPC.DEEPTHOUGHT.

[ece373172-bib-0020] Gioia, P. , and S. D. Hopper . 2017. “A New Phytogeographic Map for the Southwest Australian Floristic Region After an Exceptional Decade of Collection and Discovery.” Botanical Journal of the Linnean Society 184: 1–15.

[ece373172-bib-0021] Gomes, S. I. F. , P. Gundersen , T. M. Bezemer , et al. 2025. “Soil Microbiome Inoculation for Resilient and Multifunctional New Forests in Post‐Agricultural Landscapes.” Global Change Biology 31: e70031. 39829414 10.1111/gcb.70031

[ece373172-bib-0022] Jost, L. 2006. “Entropy and Diversity.” Oikos 113: 363–375.

[ece373172-bib-0023] Kuhn, M. 2015. “Caret: Classification and Regression Training.” Astrophysics Source Code Library:ascl: 1505.1003.

[ece373172-bib-0024] Lambers, H. , M. C. Brundrett , J. A. Raven , and S. D. Hopper . 2011. “Plant Mineral Nutrition in Ancient Landscapes: High Plant Species Diversity on Infertile Soils Is Linked to Functional Diversity for Nutritional Strategies.” Plant and Soil 348: 7–27.

[ece373172-bib-0025] Liddicoat, C. , S. L. Krauss , A. Bissett , et al. 2022. “Next Generation Restoration Metrics: Using Soil eDNA Bacterial Community Data to Measure Trajectories Towards Rehabilitation Targets.” Journal of Environmental Management 310: 114748. 35192978 10.1016/j.jenvman.2022.114748

[ece373172-bib-0026] Lin, H. , and S. D. Peddada . 2024. “Multigroup Analysis of Compositions of Microbiomes With Covariate Adjustments and Repeated Measures.” Nature Methods 21: 83–91. 38158428 10.1038/s41592-023-02092-7 PMC10776411

[ece373172-bib-0027] Liu, L. , P. Gundersen , T. Zhang , and J. Mo . 2012. “Effects of Phosphorus Addition on Soil Microbial Biomass and Community Composition in Three Forest Types in Tropical China.” Soil Biology & Biochemistry 44: 31–38.

[ece373172-bib-0028] Lu, J. , F. P. Breitwieser , P. Thielen , and S. L. Salzberg . 2017. “Bracken: Estimating Species Abundance in Metagenomics Data.” PeerJ Computer Science 3: e104. 10.7717/peerj-cs.104 PMC12016282 40271438

[ece373172-bib-0029] Lu, J. , N. Rincon , D. E. Wood , et al. 2022. “Metagenome Analysis Using the Kraken Software Suite.” Nature Protocols 17: 2815–2839. 36171387 10.1038/s41596-022-00738-y PMC9725748

[ece373172-bib-0030] Mason, C. N. , S. Shahar , K. K. Beals , et al. 2023. “Taxonomic and Functional Restoration of Tallgrass Prairie Soil Microbial Communities in Comparison to Remnant and Agricultural Soils.” FEMS Microbiology Ecology 99: fiad120. 37791391 10.1093/femsec/fiad120

[ece373172-bib-0031] Mohr, J. J. , P. A. Harrison , J. Stanhope , and M. F. Breed . 2022. “Is the Genomics ‘Cart’ Before the Restoration Ecology ‘Horse’? Insights From Qualitative Interviews and Trends From the Literature.” Philosophical Transactions of the Royal Society B 377: 20210381. 10.1098/rstb.2021.0381 PMC9234818 35757881

[ece373172-bib-0032] Myers, N. , R. A. Mittermeier , C. G. Mittermeier , G. A. Da Fonseca , and J. Kent . 2000. “Biodiversity Hotspots for Conservation Priorities.” Nature 403: 853–858. 10706275 10.1038/35002501

[ece373172-bib-0033] Nkongolo, K. , and R. Narendrula‐Kotha . 2020. “Advances in Monitoring Soil Microbial Community Dynamic and Function.” Journal of Applied Genetics 61: 249–263. 32062778 10.1007/s13353-020-00549-5

[ece373172-bib-0034] Oksanen, J. , F. G. Blanchet , R. Kindt , et al. 2013. “*Package ‘vegan’. Community Ecology Package, Version 2:1‐295*.” R Foundation for Statistical Computing, Vienna, Austria.

[ece373172-bib-0035] Oliverio, A. M. , A. Bissett , K. Mcguire , K. Saltonstall , B. L. Turner , and N. Fierer . 2020. “The Role of Phosphorus Limitation in Shaping Soil Bacterial Communities and Their Metabolic Capabilities.” MBio 11: 10‐1128. 10.1128/mbio.01718-01720. PMC7593963 33109755

[ece373172-bib-0036] Overbeek, R. , T. Disz , and R. Stevens . 2004. “The SEED: A Peer‐to‐Peer Environment for Genome Annotation.” Communications of the ACM 47: 46–51.

[ece373172-bib-0037] Pang, F. , Q. Li , M. K. Solanki , Z. Wang , Y.‐X. Xing , and D.‐F. Dong . 2024. “Soil Phosphorus Transformation and Plant Uptake Driven by Phosphate‐Solubilizing Microorganisms.” Frontiers in Microbiology 15: 1383813. 38601943 10.3389/fmicb.2024.1383813 PMC11005474

[ece373172-bib-0038] Parkhurst, T. , S. M. Prober , R. J. Hobbs , and R. J. Standish . 2022. “Global Meta‐Analysis Reveals Incomplete Recovery of Soil Conditions and Invertebrate Assemblages After Ecological Restoration in Agricultural Landscapes.” Journal of Applied Ecology 59: 358–372.

[ece373172-bib-0039] Parkhurst, T. , R. J. Standish , and S. M. Prober . 2022. “P Is for Persistence: Soil Phosphorus Remains Elevated for More Than a Decade After Old Field Restoration.” Ecological Applications 32: e2547. 35080806 10.1002/eap.2547

[ece373172-bib-0040] Peddle, S. D. , C. Cando‐Dumancela , S. L. Krauss , C. Liddicoat , A. Sanders , and M. F. Breed . 2024. “Agricultural Land‐Use Legacies Affect Soil Bacterial Communities Following Restoration in a Global Biodiversity Hotspot.” Biological Conservation 290: 110437.

[ece373172-bib-0041] Peddle, S. D. , R. J. Hodgson , R. J. Borrett , et al. 2025. “Practical Applications of Soil Microbiota to Improve Ecosystem Restoration: Current Knowledge and Future Directions.” Biological Reviews 100: 1–18. 39075839 10.1111/brv.13124 PMC11718600

[ece373172-bib-0042] R Core Team . 2024. R: A Language and Environment for Statistical Computing. R Foundation for Statistical Computing.

[ece373172-bib-0043] Robinson, J. M. , A. Annells , C. Cando‐Dumancela , and M. F. Breed . 2024. “Sonic Restoration: Acoustic Stimulation Enhances Plant Growth‐Promoting Fungi Activity.” Biology Letters 20: 20240295. 39353567 10.1098/rsbl.2024.0295 PMC11444772

[ece373172-bib-0044] Robinson, J. M. , R. Hodgson , S. L. Krauss , et al. 2023. “Opportunities and Challenges for Microbiomics in Ecosystem Restoration.” Trends in Ecology & Evolution 38: 1189–1202. 37648570 10.1016/j.tree.2023.07.009

[ece373172-bib-0045] Robinson, J. M. , C. Liddicoat , M. Muñoz‐Rojas , and M. F. Breed . 2024. “Primer: Restoring Soil Biodiversity.” Current Biology 34: R393–R398. 38714171 10.1016/j.cub.2024.02.035

[ece373172-bib-0046] Ryan, M. H. , S. Ehrenberg , R. G. Bennett , and M. Tibbett . 2009. “Putting the P in Ptilotus: A Phosphorus‐Accumulating Herb Native to Australia.” Annals of Botany 103: 901–911. 19213796 10.1093/aob/mcp021 PMC2707898

[ece373172-bib-0047] Silva, G. G. Z. , K. T. Green , B. E. Dutilh , and R. A. Edwards . 2015. “SUPER‐FOCUS: A Tool for Agile Functional Analysis of Shotgun Metagenomic Data.” Bioinformatics 32: 354–361. 26454280 10.1093/bioinformatics/btv584 PMC4734042

[ece373172-bib-0048] Suding, K. N. , K. L. Gross , and G. R. Houseman . 2004. “Alternative States and Positive Feedbacks in Restoration Ecology.” Trends in Ecology & Evolution 19: 46–53. 16701225 10.1016/j.tree.2003.10.005

[ece373172-bib-0049] Sun, S. , and B. D. Badgley . 2019. “Changes in Microbial Functional Genes Within the Soil Metagenome During Forest Ecosystem Restoration.” Soil Biology and Biochemistry 135: 163–172.

[ece373172-bib-0050] Toledo, R. M. , R. F. Santos , L. Baeten , M. P. Perring , and K. Verheyen . 2018. “Soil Properties and Neighbouring Forest Cover Affect Above‐Ground Biomass and Functional Composition During Tropical Forest Restoration.” Applied Vegetation Science 21: 179–189.

[ece373172-bib-0051] Turley, N. E. , L. Bell‐Dereske , S. E. Evans , and L. A. Brudvig . 2020. “Agricultural Land‐Use History and Restoration Impact Soil Microbial Biodiversity.” Journal of Applied Ecology 57: 852–863.

[ece373172-bib-0052] Van Der Heyde, M. , M. Bunce , and P. Nevill . 2022. “Key Factors to Consider in the Use of Environmental DNA Metabarcoding to Monitor Terrestrial Ecological Restoration.” Science of the Total Environment 848: 157617. 35901901 10.1016/j.scitotenv.2022.157617

[ece373172-bib-0053] Wang, Y. , H. Bing , D. L. Moorhead , et al. 2024. “Bacterial Community Structure Modulates Soil Phosphorus Turnover at Early Stages of Primary Succession.” Global Biogeochemical Cycles 38: e2024GB008174.

[ece373172-bib-0054] Watson, C. D. , M. G. Gardner , R. J. Hodgson , C. Liddicoat , S. D. Peddle , and M. F. Breed . 2022. “Global Meta‐Analysis Shows Progress Towards Recovery of Soil Microbiota Following Revegetation.” Biological Conservation 272: 109592.

[ece373172-bib-0055] Wood, D. E. , J. Lu , and B. Langmead . 2019. “Improved Metagenomic Analysis With Kraken 2.” Genome Biology 20: 257. 31779668 10.1186/s13059-019-1891-0 PMC6883579

[ece373172-bib-0056] Yao, Q. , Z. Li , Y. Song , et al. 2018. “Community Proteogenomics Reveals the Systemic Impact of Phosphorus Availability on Microbial Functions in Tropical Soil.” Nature Ecology & Evolution 2: 499–509. 29358607 10.1038/s41559-017-0463-5

[ece373172-bib-0057] Zhu, L. , Y. Chen , R. Sun , et al. 2023. “Resource‐Dependent Biodiversity and Potential Multi‐Trophic Interactions Determine Belowground Functional Trait Stability.” Microbiome 11: 95. 37127665 10.1186/s40168-023-01539-5 PMC10150482

