# Supplementary material for: Soil Microbial Functions Indicate Persistent Agricultural Legacies and Potential Alternative States Following Restoration Plantings
Source: Ecol Evol. 2026 Feb 26;16(3):e73172. doi: 10.1002/ece3.73172 (PMC12946517; doi:10.1002/ece3.73172)
Supplement: Supplementary file 1 — Data S1: ece373172‐sup‐0001‐Supinfo.docx. [file ECE3-16-e73172-s001.docx]

*Supplementary Information for the article:*

**Soil microbial functions indicate persistent agricultural legacies and alternative stable states following restoration plantings**

Shawn D. Peddle^1^, Christian Cando-Dumancela^1^, Sofie Costin^1^, Tarryn Davies^1^,
Michael P. Doane^1^, Robert A. Edwards^1^, Riley J. Hodgson^1^, Siegfried L. Krauss^2,3^, Craig Liddicoat^1^, Martin F. Breed^1^

^1^College of Science and Engineering, Flinders University, Bedford Park, SA, Australia. ^2^The University of Western Australia, Crawley, WA, Australia.

^3^Kings Park Science, Department of Biodiversity, Conservation and Attractions, Perth, WA, Australia.

**Corresponding author:** Shawn Peddle, Tel: +61 08 8201 2113, Email: [shawn.peddle@flinders.edu.au](mailto:shawn.peddle@flinders.edu.au) College of Science and Engineering, Flinders University, Bedford Park, SA, Australia

**Author contributions: Shawn D. Peddle:** Conceptualisation; investigation; data curation; formal analysis; writing – original draft preparation; writing – review and editing. **Christian Cando-Dumancela:** Data curation; resources; writing – review and editing. **Sofie Costin:** Resources; visualisation; writing – editing and review; **Tarryn Davies:** Resources; writing – review and editing; **Michael P. Doane:** Resources; writing – review and editing. **Robert A. Edwards:** Resources; funding acquisition, supervision, writing – review and editing. **Riley J. Hodgson:** Formal analysis; writing – review and editing. **Siegfried L. Krauss:** Conceptualisation; resources; supervision; writing – review and editing. **Craig Liddicoat:** Formal analysis, supervision, writing – review and editing. **Martin F. Breed:** Conceptualisation; resources; funding acquisition; supervision; writing – review and editing.

**ORCIDs:** SDP 0000-0003- 3464-3058, CCD 0000-0003-0186-0056, SLK 0000-0002-7280-6324, CL 0000-0002-4812-7524, MFB 0000-0001-7810-969

**Figure S1** Relative abundance stacked bar plots of (A) Phylum level (taxonomic) relative abundance and (B) subsystem 1 (function) relative abundance. Bolded labels indicate levels that significantly differ (permuted ANOVA, *p* < 0.05) across land condition.

**Figure S2** Relative abundance stacked bar plot of phosphorus metabolism functions at subsystem 3. No subsystem 3 phosphorus metabolism functional groups differed (permuted ANOVA, *p* > 0.05) across land condition.

**Figure S3** Relative abundance staked bar plot of Iron acquisition and metabolism functions at subsystem 3. Bolded functions significantly differ (permuted ANOVA, *p* < 0.05) across land condition.

**Figure S4** Relative abundance staked bar plot of sulphur metabolism functions at subsystem 3. Bolded functions significantly differ (permuted ANOVA, *p* < 0.05) across land condition.

**Figure S5** Relative abundance staked bar plot of amino acids and derivatives functions at subsystem 3. Bolded functions significantly differ (permuted ANOVA, *p* < 0.05) across land condition.

**Figure S6** Relative abundance staked bar plot of carbohydrates functions at subsystem 2. No functions significantly differed (permuted ANOVA, *p* > 0.05) across land condition.

**Figure S7** Relative abundance staked bar plot of clustering-based subsystems functions at subsystem 2. Bolded functions significantly differ (permuted ANOVA, *p* < 0.05) across land condition.

**Figure S8** Relative abundance staked bar plot of DNA metabolism functions at subsystem 3. Bolded functions significantly differ (permuted ANOVA, *p* < 0.05) across land condition.

**Figure S9** Relative abundance staked bar plot of regulation and cell signalling functions at subsystem 3. Bolded functions significantly differ (permuted ANOVA, *p* < 0.05) across land condition.

**Figure S10** Relative abundance staked bar plot of nitrogen metabolism functions at subsystem 3. Bolded functions significantly differ (permuted ANOVA, *p* < 0.05) across land condition.

**Table S1** Summary table of read depth (mean ± SD) and numbers of species and functions (mean ± SD) for each land condition. Taxonomy refers to the cleaned (Eukaryotes and taxa not assigned at the phylum level removed) taxonomic dataset and functions refers to the full non-normalised functions dataset. Number of species is at the species level and functions is at the functional process subsystem level.

| Land Condition | Taxonomy Read Depth (mean ± SD) | Functions Read Depth (mean ± SD) | Number of Species (mean ± SD) | Number of Functions (mean ± SD) |
| --- | --- | --- | --- | --- |
| Degraded | 2,782,645.67 ± 1,046,248.49 | 3,218,295.33 ± 1,104,679.42 | 10,294.50 ± 336.68 | 23,791.17 ± 1,123.18 |
| Regenerated | 3,249,465.25 ± 664,193.01 | 3,772,910.00 ± 606,873.18 | 10,433.50 ± 253.24 | 24,527.50 ± 774.98 |
| Revegetated | 3,482,459.50 ± 428,974.71 | 3,151,039.40 ± 1,290,283.37 | 10,388.83 ± 174.74 | 24,320.83 ± 479.92 |
| Remnant | 2,751,344.60 ± 1,229,966.92 | 3,750,099.67 ± 511,453.73 | 10,278.00 ± 362.56 | 23,533.40 ± 1,641.90 |

**Table S2** Table of mean (± S.D.) effective number of species or functions (alpha diversity) in each land condition for each analysed group (taxonomy, all functions, or subsystem 1 functional level). Land condition levels that do not share a subscript letter indicate significant (ANOVA, *p* < 0.05) differences between land conditions within the group.

| Group | Land Condition | Effective no. species/functions (mean ± S.D.) |
| --- | --- | --- |
| Taxonomy | Degraded | 2500.00 ± 265.11_a_ |
|  | Regenerated | 2450.89 ± 95.28_a_ |
|  | Revegetated | 2196.32 ± 252.89_a_ |
|  | Remnant | 2419.69 ± 102.89_a_ |
| All functions | Degraded | 3622.64 ± 63.01_a_ |
|  | Regenerated | 3688.24 ± 82.44_a_ |
|  | Revegetated | 3639.05 ± 44.81_a_ |
|  | Remnant | 3692.98 ± 48.50_a_ |
| Phosphorus metabolism | Degraded | 37.78 ± 0.53_a_ |
|  | Regenerated | 39.01 ± 1.55_ab_ |
|  | Revegetated | 37.99 ± 1.00_a_ |
|  | Remnant | 40.81 ± 1.47_b_ |
| Iron acquisition and metabolism | Degraded | 51.90 ± 1.77_a_ |
|  | Regenerated | 52.68 ± 3.15_a_ |
|  | Revegetated | 50.33 ± 2.85_a_ |
|  | Remnant | 51.69 ± 3.48_a_ |
| Nitrogen metabolism | Degraded | 35.77 ± 2.68_a_ |
|  | Regenerated | 35.93 ± 2.82_a_ |
|  | Revegetated | 32.77 ± 2.08_a_ |
|  | Remnant | 34.50 ± 3.61_a_ |
| Sulphur acquisition and metabolism | Degraded | 87.88 ± 2.87_a_ |
|  | Regenerated | 91.29 ± 3.73_a_ |
|  | Revegetated | 87.66 ± 1.86_a_ |
|  | Remnant | 90.57 ± 3.81_a_ |
| Amino acids and derivatives | Degraded | 431.86 ± 7.24_a_ |
|  | Regenerated | 439.54 ± 10.46_a_ |
|  | Revegetated | 430.05 ± 5.73_a_ |
|  | Remnant | 438.39 ± 11.21_a_ |
| Carbohydrates | Degraded | 616.92 ± 13.79_a_ |
|  | Regenerated | 631.50 ± 18.62_a_ |
|  | Revegetated | 618.48 ± 10.74_a_ |
|  | Remnant | 633.10 ± 14.93_a_ |
| Clustering-based subsystems | Degraded | 238.25 ± 6.74_a_ |
|  | Regenerated | 245.77 ± 9.88_a_ |
|  | Revegetated | 241.08 ± 5.45_a_ |
|  | Remnant | 243.80 ± 4.40_a_ |
| DNA metabolism | Degraded | 2380.04 ± 256.24_ab_ |
|  | Regenerated | 2339.50 ± 86.20_ab_ |
|  | Revegetated | 2094.29 ± 246.05_a_ |
|  | Remnant | 2312.63 ± 101.58_b_ |
| Regulation and cell signalling | Degraded | 85.89 ± 3.16_a_ |
|  | Regenerated | 87.86 ± 5.46_a_ |
|  | Revegetated | 87.93 ± 3.71_a_ |
|  | Remnant | 87.74 ± 6.81_a_ |

**Table S3** Permutational multivariate analysis of variance (PERMANOVA) results from tests assessing differences in either community (taxonomy) or functional (all others) compositions (beta diversity) across our four land conditions and six sites. Bolded values indicate significant differences (*p* < 0.05) across either land condition or site.

| Group | *F* | R^2^ | Land Condition  *p* | *F* | R^2^ | Site  *p* |
| --- | --- | --- | --- | --- | --- | --- |
| Taxonomy | 2.242 | 0.283 | **0.009** | 1.703 | 0.362 | **0.029** |
| All functions | 2.015 | 0.262 | **0.015** | 1.977 | 0.397 | **0.006** |
| Phosphorus metabolism | 1.683 | 0.229 | 0.079 | 2.917 | 0.493 | **0.001** |
| Iron acquisition and metabolism | 1.730 | 0.234 | 0.07 | 2.607 | 0.465 | **0.002** |
| Nitrogen metabolism | 2.057 | 0.266 | **0.041** | 2.244 | 0.428 | **0.011** |
| Sulphur acquisition and metabolism | 2.025 | 0.263 | **0.021** | 1.754 | 0.369 | **0.022** |
| Amino acids and derivatives | 1.999 | 0.261 | **0.023** | 1.939 | 0.393 | **0.008** |
| Carbohydrates | 1.631 | 0.224 | 0.076 | 2.197 | 0.423 | **0.003** |
| Clustering-based subsystems | 2.493 | 0.305 | **0.011** | 1.750 | 0.368 | **0.049** |
| DNA metabolism | 2.051 | 0.266 | **0.02** | 1.495 | 0.333 | 0.083 |
| Regulation and cell signalling | 2.655 | 0.319 | **0.006** | 1.372 | 0.314 | 0.158 |

**Table S4** Significant log fold change (LFC) values for differently abundant phosphorus metabolism functions from pairwise comparisons of each land condition all to the remnant condition.

| Function | Comparison | LFC |
| --- | --- | --- |
| Phosphonate utilization as. acetyltransferase | Revegetated | -0.930 |
| Phosphonate utilization as. acetyltransferase | Regenerated | -0.212 |
| Phosphonate utilization as. acetyltransferase | Degraded | -0.779 |
| Ribose 1,5 biphosphate phosphokinase | Revegetated | -0.883 |
| Ribose 1,5 biphosphate phosphokinase | Regenerated | -0.560 |
| Ribose 1,5 biphosphate phosphokinase | Revegetated | -0.876 |
